# The Complete Sequence of the Mitochondrial Genome of *Butomus umbellatus* – A Member of an Early Branching Lineage of Monocotyledons

**DOI:** 10.1371/journal.pone.0061552

**Published:** 2013-04-24

**Authors:** Argelia Cuenca, Gitte Petersen, Ole Seberg

**Affiliations:** Botanical Garden, Natural History Museum of Denmark, University of Copenhagen, Copenhagen K, Denmark; Centro de Investigación y de Estudios Avanzados del IPN, Mexico

## Abstract

In order to study the evolution of mitochondrial genomes in the early branching lineages of the monocotyledons, i.e., the Acorales and Alismatales, we are sequencing complete genomes from a suite of key taxa. As a starting point the present paper describes the mitochondrial genome of *Butomus umbellatus* (Butomaceae) based on next-generation sequencing data. The genome was assembled into a circular molecule, 450,826 bp in length. Coding sequences cover only 8.2% of the genome and include 28 protein coding genes, four rRNA genes, and 12 tRNA genes. Some of the tRNA genes and a 16S rRNA gene are transferred from the plastid genome. However, the total amount of recognized plastid sequences in the mitochondrial genome is only 1.5% and the amount of DNA transferred from the nucleus is also low. RNA editing is abundant and a total of 557 edited sites are predicted in the protein coding genes. Compared to the 40 angiosperm mitochondrial genomes sequenced to date, the GC content of the *Butomus* genome is uniquely high (49.1%). The overall similarity between the mitochondrial genomes of *Butomus* and *Spirodela* (Araceae), the closest relative yet sequenced, is low (less than 20%), and the two genomes differ in size by a factor 2. Gene order is also largely unconserved. However, based on its phylogenetic position within the core alismatids *Butomus* will serve as a good reference point for subsequent studies in the early branching lineages of the monocotyledons.

## Introduction

The mitochondrial genomes of embryophytes or land plants are renowned among eukaryotes for their astonishing complexity, and evolutionary plasticity seems to prevail among angiosperms in particular. The 40 complete angiosperm mitochondrial genomes sequenced as of December 3^rd^ 2012 (GenBank) vary enormously in size, from only ca. 0.2 Mb in *Brassica* [1] to ca. 11.3 Mb in *Silene conica* [2], and in mapping structure. The majority of mitochondrial genomes can be mapped to a single circular molecule, except in *Silene* and *Cucumis* where they were mapped to several individual chromosomes [3], [4]. Even when the genomes can be mapped to a master circle [5] this structure is not stabile and sub-molecules are formed, facilitated by recombination across repeated sequences [6]. Consequently, gene order is remarkably unconserved even between closely related species e.g., [1], [7].

Other peculiarities of angiosperm mitochondrial genomes include substantial variation in gene content, frequent intracellular import of foreign sequences from the plastid and the nuclear genome, export of sequences to the nuclear genome, incorporation of reverse transcribed gene sequences known as “processed paralogs”, frequent RNA editing of both coding and non-coding sequences, vast substitution rate heterogeneity, and postulated ability to import and export sequences horizontally, i.e. across species boundaries (see [4] for a recent review). A striking aspect of mitochondrial evolution in angiosperms is the fact that most of the variable features mentioned above not just vary across the group as a whole but even among closely related taxa; e.g., species of *Silene* [2], [8], [9], *Brassica* [1], *Pelargonium* [10], [11], and *Plantago* [11], [12].

Given the huge structural differences sometimes even among closely related species and the limited number of completely sequenced genomes, it is not surprising that the evolutionary mechanisms facilitating these changes are largely unknown. Thus, a much wider taxonomic sampling is needed in order to throw light on other aspects of mitochondrial evolution. Most mitochondrial genomes sequenced to date are from agronomically important species and the taxonomic distribution of the 40 angiosperm genomes is heavily skewed: 12 genomes are from monocotyledons, but of these 10 are grasses, and the remaining 28 genomes are from 14 families of eudicots. Thus, mitochondrial structure and evolution in the early branching angiosperm lineages remains completely unknown though the sequence of the mitochondrial genome of *Amborella*, assumed to be the sister group to all other angiosperms, may be under way [13]. In the monocotyledons the only two non-grass genomes are from *Spirodela*, duckweed, (Araceae) [14] and *Phoenix*, date palm, (Arecaceae) [15].

The present paper is intended as the first in a series devoted to the study of the evolution of whole mitochondrial genomes in the early branching lineages of monocotyledons, i.e., the Acoraceae and the Alismatales. According to the APG III [16], Acoraceae is the sister group to all other monocotyledons and within these Alismatales is the sister group to the remaining. Studies of selected genes have previously shown evolutionary anomalies in parts of the Alismatales [17]–[19]. In the present paper, we describe the first completely sequenced genome from a member of the core alismatids, i.e., Alismatales, excl. Araceae and Tofieldiaceae. Based on a single genome only few evolutionary questions can be addressed. However, due to its the phylogenetic position within the core alismatids [17], it will serve as an appropriate reference for subsequent comparisons.

## Materials and Methods

### Mitochondrial Genome Sequencing

Fresh plant material was collected from a single individual (voucher: Seberg et al. C2457 (C)) at St.Vejleå, Ishøj in Denmark (N 55°37.496′ E 12°21.939′). No specific permits were required for the collection of this material. The species is not protected by Danish law and it is collected in a public area where no permits are needed. Intact mitochondria were isolated by centrifugation following a modified protocol of Triboush et al. [20], and using DNAase I to digest nuclear and other DNA contaminants. Mitochondrial DNA was extracted using CTAB and a regular chloroform-isoamilic DNA isolation protocol. Whole genome amplifications were carried out using the repli-g kit (Qiagen), following the manufacturer’s protocol. To check the identity of the DNA obtained by whole genome amplifications, we attempted to amplify four partial mitochondrial genes (*ccmB, mtt2, nad1*, and *nad5*), one partial plastid gene (*rbcL*), and the nuclear ITS region. Whereas all mitochondrial amplifications were successful, the plastid and nuclear amplifications were not indicating that the extraction and subsequent whole genome amplification procedure provided clean mitochondrial DNA.

A standard 454 FLX (Roche, USA) shoot gun library was constructed and sequenced in a quarter of a GS PicoTiterPlate according to the manufacturer’s instructions at the National High-throughput DNA Sequencing Centre, University of Copenhagen.

### Sequence Assembly

A total of 87,048 sequences (average size 207 nt) were assembled in Newbler 2.3 (454 Life Sciences Corp, CT, USA) using default settings. This resulted in 572 contigs ranging from 100 to 56,599 nt and 76 were longer than 500 nt (average size 6238 nt). The contigs were extended by blasting the last ca. 75 nt of each contig border against a database of all raw 454 sequence reads. This allowed us to determine the borders of duplications and to identify reads of adjacent contigs. All BLAST analyses were done using the BLASTN program in the stand-alone BLAST ver. 2.2.21 (ftp://ftp.ncbi.nlm.nih.gov/blast/executables/blast/LATEST/). In addition, the consensus sequence of each contig was used as seed sequences and extended using the Short Sequence Assembly by K-mer search and 3′ read Extension program, SSAKE ver. 3.5 [21], with parameters -m 15 -o 2 -r 0.6 -p 0 -t 0 -v 1. In cases where contig extension was not possible, primers were designed and gap closure was done by combinatorial PCR [22] and regular Sanger sequencing. Sequencing data is deposited in DRYAD, DOI: 10.5061/dryad.42gc4. The assembled sequence is deposited in GenBank under accession number KC208619.

### Sequence Analyses

Coding regions were identified by BLASTX searches performed against a local database including amino acid sequences for all coding genes from 20 plant mitochondrial genomes available in GenBank The exact gene and exon boundaries were determined by alignment of homologous genes from available annotated plant mitochondrial genes. Similarly, rRNA and tRNA genes were identified by BLASTN searches against a local database including all rRNA genes and a database including all tRNA genes from 20 available land plant mitochondrial genomes. tRNAs were annotated using tRNAscan-SE [23], [24]. To identify potential regions of plastid origin the mitochondrial genome of *Butomus* was blasted against a database of 20 land plant plastid genomes, including *Lemna* (Araceae) and *Acorus* (Acoraceae), both closely related to *Butomus*. Only sequences >50 bp and with a similarity score higher than 80% were considered. To identify potential regions of nuclear origin we primarily searched for repetitive elements using the Repbase Update repetitive element data base [25], but in addition a BLASTN search of three long intergenic regions of the mitochondrial genome were performed against the GenBank Nucleotide Collection filtering for plastid and mitochondrial sequences. To test the overall similarity between the entire *Butomus* mitochondrial genome and other complete angiosperm mitochondrial genomes a BLASTN search was performed using complete mitochondrial sequences as input.

### Phylogenetic Analyses

Sequences of 24 mitochondrial genes from *Butomus* and 25 seed plant species, for which the complete mitochondrial genome is available, were extracted from GenBank (see Table 1). The genes include all protein coding genes present in *Butomus* except the ribosomal genes (Table 2). Alignments were generated for each individual gene using MUSCLE [26] integrated in Geneious Pro (ver. 5.3.6; Biomatters Ltd.) with default parameters and concatenated into a matrix of a total of 28,455 characters. The matrix has a few missing entries, viz. the *cox2* gene is missing in *Vigna* and the *mttB* gene is missing in *Vitis* and *Boea.* A Maximum Likelihood tree was constructed using the program PhyML [27] with a GTR substitution model and a gamma distribution of substitution rates estimated with four categories.

**Table 1:** Comparison of complete mitochondrial genomes of selected seed plants.

Species	Refseq	Length	GC^1^	Coding^2^	Protein^3^	tRNA^4^	rRNA	Introns^5^	Plastid^5^	Nuclear^5^	Repeats^6^	Edited
		(bp)	(%)	(%)	genes	genes	genes	total (*trans*)	(%)	(%)	(%)	Sites^7^
*Cycas taitungensis*	NC_010303	414,903	46.9	10.0	41	22	3	25 (5)	4.4	–	15.1	1084^8^
*Butomus umbellatum*	KC208619	450,826	49.1	8.2	28	12	4^9^	21 (5)	–	0	–	557^8^
*Spirodela polyrhiza*	NC_017840	228,493	45.7	16.2	35	18	3	21 (6)	4.1	0	–	540^8^
*Phoenix dactylifera*	NC_016740	715,001	45.1	6.3	38	22	3	24 (4)	10.3	–	2.3	592^8^
*Bambusa oldhamii*	EU365401	509,941	44.1	7.8	35	18	3	22 (6)	–	–	–	–
*Oryza sativa* ssp. *indica*	NC_007886	491,515	43.8	10.2	35	21	3	23 (6)	6.3	13.4	26	491
*Triticum aestivum*	NC_007579	452,528	44.4	11.4	33	16	3	22 (6)	3.0	–	10.1	–
*Sorghum bicolor*	NC_008360	468,628	43.7	8.2	32	19	3	22 (6)	–	–	–	–
*Tripsacum dactyloides*	NC_008362	704,100	43.9	5.9	32	17	3	22 (6)	–	–	–	–
*Zea luxurians*	NC_008333	539,368	43.9	7.2	32	16	3	22 (6)	–	–	–	–
*Zea mays* ssp. *mays*	NC_007982	569,630	43.9	8.4	32	18	3	22 (7)	4.5	–	22.9	–
*Vitis vinifera*	NC_012119	773,279	44.1	6.3	38	23	3	25 (5)	8.8	–	6.84	401^10^
*Arabidopsis thaliana*	NC_001284	366,924	44.8	11.5	31	17	3	23 (5)	1	4	7	441
*Brassica napus*	NC_008285	221,853	45.2	17.4	32	18	3	24 (5)	3.6	–	5.5	427
*Carica papaya*	NC_012116	476,890	45.1	8.6	39	20	3	24 (5)	–	–	–	–
*Citrullus lanatus*	NC_014043	379,236	45.1	10.3	38	20	3	24 (5)+1^11^	6	6.4	10	463
*Cucurbita pepo*	NC_014050	982,833	42.8	3.9	38	26	3	24 (5)	11.5	2.3	38	444
*Cucumis sativus^12^*	NC_016005	1,555,935	44.3	2.9	37	22	3	23 (5)+1^11^	4.6	1.3	36	–
*Lotus japonicas*	NC_016743	380,861	45.4	9.9	31	16	3	22 (5)	–	–	–	–
*Millettia pinnata*	NC_016742	425,718	45.0	9.7	33	17	3	23 (5)	–	–	–	–
*Vigna radiate*	NC_015121	401,262	45.1	8.8	31	14	3	22 (5)	0.5	–	–	–
*Nicotiana tabacum*	NC_006581	430,597	45.0	10.1	37	21	3	23 (6)	2.5	–	>8.1	514^8^
*Beta vulgaris*	NC_002511	368,801	43.9	11.3	30	21	3	20 (6)	2.1	3.3	10.3	357
*Silene latifolia*	NC_014487	253,413	42.6	14.5	25	9	3	19 (6)	1	–	–	287
*Daucus carota*	NC_017855	281,132	45.4	18.4	34	17	3	24 (5)	2	–	46	–
*Boea hygrometrica*	NC_016741	510,519	43.3	7.6	30	23	6^13^	23 (5)	10.5	–	1.5	–
*Ricinus communis*	NC_015141	502,773	45.0	7.6	37	16	3	24 (5)	–	–	–	–

**Table 2:** Gene content of the mitochondrial genome of *Butomus umbellatus*.

**Genes of mitochondrial origin**	
Complex I (NADH dehydrogenase)	*nad1*, *nad2*, *nad3*, *nad4^1^*, *nad4L*, *nad5*, *nad6*, *nad7, nad9*
Complex II (succinate dehydrogenase)	–
Complex III (cytochrome C reductase)	*cob*
Complex IV (cytochrome C oxidase)	*cox1*, *cox2^2^*, *cox3*
Complex V (ATP Synthase)	*atp1*, *atp4*, *atp6*, *atp8*, *atp9*
Cytochrome C biogenesis	*ccmB*, *ccmC^3^*, *ccmFc*, *ccmFn*
Other genes	*matR*, *mttB*
Large subunit ribosomal proteins	–
Small subunit ribosomal proteins	*rps1*, *rps3*, *rps7*, *rps12^4^*
Transfer RNAs	*trnC-GCA, trnD-GUC*, *trnE-UUC^5^, trnK-CUU*, *trnM-CAU^6^, trnQ-UUG, trnY-GUA*
Ribosomal RNAs	*rrn5, rrn18, rrn26*
Pseudogenes, partial	*sdh4-ψ*, *rpl16-ψ*
**Genes of plastid origin**	
Ribosomal RNAs	*rrn16*
Transfer RNAs	*trnA-UGC, trnH-GUG^7^*, *trnI-GAU, trnK-UUU, trnW-CCA*

To investigate substitution rate diversity among rRNA genes individual alignments were also done for each of the three rRNAs universally present in the plant mitochondria (*rrn5*, *rrn18*, and *rrn26*) using MUSCLE integrated in Geneious Pro. Alignments were performed for the same taxa as above, except that *Boea* was excluded because its *rrn18* sequence appear quite different and could not be aligned easily. The phylogenetic tree resulting from the analysis of the 24 protein coding genes was used to estimate the substitution rate of the three rRNAs. To estimate substitution rates, the JC+G model was used for *rrn5* (119 bp), the TPM1+G (K81) model for *rrn18* (2553 bp), and the GTR+G model for *rrn26* (4498 bp), as suggested by jModelTest 0.1.1 [28]. All substitution rate were calculated using the program PAML 4.3 [29].

## Results and Discussion

### The Mitochondrial Genome of *Butomus*

**Figure 1:**
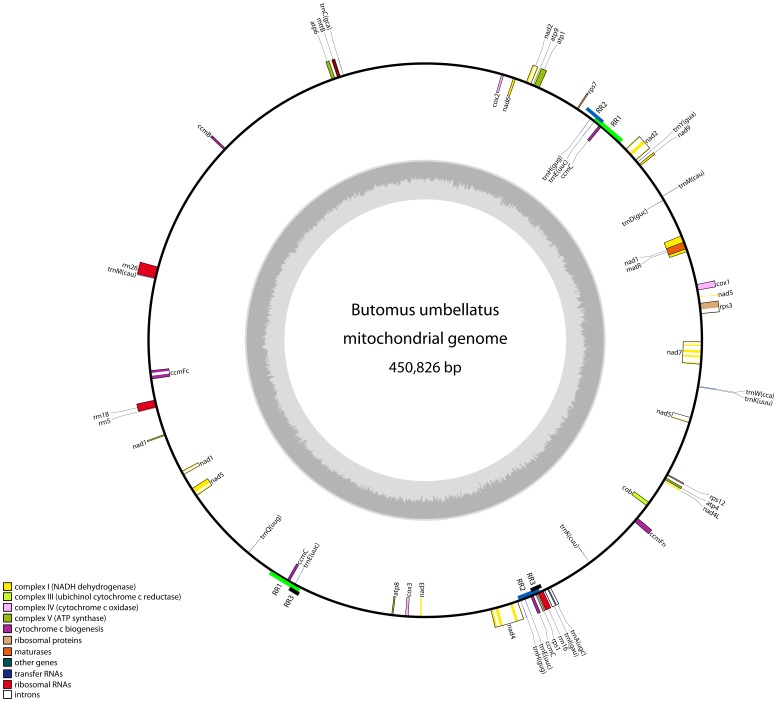
Circular map of the mitochondrial genome of *Butomus umbellatus*. Known protein coding, rRNA, and tRNA genes are shown and color coded as indicated in the lower left corner. Genes shown outside and inside of the circle are transcribed clockwise and counter clockwise, respectively. Repeats longer than 1 kb are shown as colored bars named RR1-RR3. The location of repeat units shown inside or outside of the circle is purely practical; information on directionality is given in Table 4. The inner circle shows GC content. The figure was created using OGDRAW [59].

A total of 63,056 individual sequences, corresponding to 72% of the total number of sequences obtained, were assembled into a circular molecule of 450,826 bp (Fig. 1; GenBank acc. no. KC208619) giving an average coverage of 32×. This circular molecule correspond to the so-called master circle [5] containing all mitochondrial genes of *Butomus*.

With a total genome size of ca. 451 kb the mitochondrial genome of *Butomus* is almost twice as large as the ca. 228 kb genome of *Spirodela* [14], the only other genome from the Alismatales sequences so far, but closer in size to the grass genomes ranging from ca. 453–704 kb (Table 1).

The nucleotide composition of the *Butomus* genome has the highest GC content, 49.1%, reported so far (Table 1). In other seed plants the GC% content ranges from ca. 43–47% (Table 1). Thus, the three highest percentages are found in *Cycas*, *Butomus*, and *Spirodela,* which vaguely suggest a decline in GC content during angiosperm evolution.

Coding regions constitute only 8.2% of the *Butomus* genome and with a total gene content of 28 protein-coding genes, 12 tRNA genes, and 3 (+1 CP) rRNA genes (Table 1, 2) the content of coding DNA both in terms of coverage and actual gene numbers is relatively low but not unusual compared to other angiosperms (Table 1). The genes are very unevenly distributed across the circular genome (Fig. 1). Three regions of 33–41 kb are completely devoid of genes or any other recognizable features and in a region spanning ca. 113 kb, i.e. approximately one quarter of the genome, only four genes are located (Fig. 1). Compared to the *Spirodela* genome, gene order is largely unconserved as reported from other comparative genome studies e.g., [1], [7].

Based on BLAST searches the entire mitochondrial genome of *Butomus* has the highest similarity to the ca. 526 kb genome of *Mimulus* [30], which matches 23% of the *Butomus* mitochondrial genome. As the coding sequences of each genome take up only ca. 6 and 5%, respectively (Table 1), similarity clearly extends into the non-coding regions, thus suggesting some level of sequence conservation in those regions of the genome. It might have been expected that the genomes of *Butomus* and *Spirodela*, being each others closest sequenced relatives, would have been most similar, but with only 19% overall similarity this is not so. As the *Spirodela* genome is approximately half the size of the *Butomus* genome, it may be assumed that sequence loss has reduced overall genome similarity. The overall similarity of the *Butomus* genome and the 715 kb mitochondrial genome of *Phoenix*, the only other non-grass monocotyledon, is 22%. The total amount of sequence of the *Butomus* genome being similar to other Spermatophyta mitochondrial genomes is approximately 30%. Thus, the majority of the genome is composed of so far unique sequences.

The mitochondrial genome of angiosperms is known to include intergenomic transferred DNA from both the plastid and the nuclear genome [4], but only ca. 1.5% of the *Butomus* genome may be of plastid origin and an even tinier fraction of nuclear origin (see below).

### Plastid DNA Insertions

The mitochondrial genome of vascular plants is known to import fragments of DNA originating from the plastid genome [4], [31]. In *Butomus* we detected ten fragments ranging in size from 63 to 4.897 bp (fragment less than 50 bp were not considered) with high similarity to plastid sequences (Table 3). These fragments, a total of 6.928 bp, constitute only 1,5% of the entire mitochondrial genome – considerably less than most other genomes (Table 1).

**Table 3:** Sequences of plastid origin in mitochondrial genome of *Butomus umbellatus*.

Start	End	Length (bp)	Sequence characteristics
34.784	34.889	106	*ycf2-ndhB* intergenic region
47.701	48.603	903	*ndhK*+*ndhC*, partial
62.015	62.099	85	*petD*, partial^1^
171.200	171.359	160	*psbD*, partial
215.497	215.653	157	*rpoB*, partial
249.922	249.992	71	*psbD*, partial
299.456	299540	85	*petD*, partial^1^
368.045	372.941	4.897	*rrn16*, *trnA(ugc), trnI(gau)*
434.652	434.714	63	*rps3*, partial
438.532	438.932	401	*trnK(uuu)* 2, *trnW(cca)*
		**6.928**	

1 Repeated sequence.

2 *trnP(ugg)* in CP genomes, *trnK(uuu)* in *Butomus.*

Six fragments are relatively short (63–160 bp) and include partial and apparently degenerated sequence of protein coding genes (*petD* X 2, *psbD* X 2, *rpoB*, *rps3*), but no flanking non-coding sequence, suggesting that these sequences could have been inserted through reverse transcription. A 903 bp region seems to include the entire, but degenerate, *ndhK* gene plus a small fraction of the *ndhC* gene. In plastid genomes of most land plants *ndhK* and *ndhC* have shortly overlapping reading frames (e.g., 10 bp in *Elodea*, GenBank acc. no. JQ310743) and are co-transcribed [32] making reverse transcription possible even for this fragment.

The remaining fragments (106, 401, 4.897 bp) include primarily non-coding DNA, but also tRNAs and rRNA, suggesting another mode of transfer and insertion. The longer fragment (4.897 bp) has very high similarity to a region of the plastid inverted repeat region including 16S rRNA and two tRNA genes (*trnA(ugc)*, *trnI(gau)*). This plastid region cannot be found in any other mitochondrial genome suggesting recent transfer. In contrast, the 903 bp fragment (including *ndhK* and *ndhC*) and the 401 bp fragment (including two *trnK(uuu)* and *trnW(cca)*), or substantial parts of the fragments, can be recognized in *Phoenix*, *Spirodela*, and most, but not all, grasses and eudicots suggesting more ancient transfer events followed by degeneration.

### Nuclear DNA Insertions

The mitochondrial genome may also integrate DNA from the nuclear genome [4], [33]. However, identification of nuclear sequences may be problematic due to sequence changes over time, a very sparse record of fully or even just partially sequenced nuclear genomes, and because sequence transfer between the nuclear and mitochondrial genomes occurs in both directions making it difficult to determine the directionality of transfer events of seemingly featureless DNA sequences [4], [33]. Presence of DNA sequences similar to nuclear repetitive elements in mitochondrial genomes has been used as positive evidence of nuclear DNA transfer e.g., [7], [34], [35].

A similarity search of the mitochondrial genome of *Butomus* against the Repbase Update repetitive element data base [25] revealed very few good matches (>80% similarity of sequences >50 bp long). Most of these (15) were even very short (<60 bp), the remaining just 71, 75, and 126 bp long, respectively, and in total adding up to only 1076 bp. It is questionable whether these short sequences are indeed remnants of sequences transferred from the nuclear genome, but even if they are they constitute only 0.2% of the mitochondrial genome. This low percentage is consistent with the lack of repetitive elements in the mitochondrial genome of *Spirodela* [14]. As a supplement to the Repbase Update search we conducted a BLASTN search against GenBank sequences filtering for chloroplast and mitochondrial sequences. In this search we only used the three larger fragments of the *Butomus* mitochondrial genome devoid of recognizable features (see above) covering approximately a quarter of the genome. The search did not reveal any good matches to sequences of unequivocal nuclear origin. As we did not perform similar searches with the remaining intergenic regions of the mitochondrial genome we cannot rule out the possibility that some of these may include sequences of nuclear origin, but we assume that the general content of nuclear DNA in the *Butomus* mitochondrial genome is very low indeed.

### Protein Coding Genes

**Figure 2:**
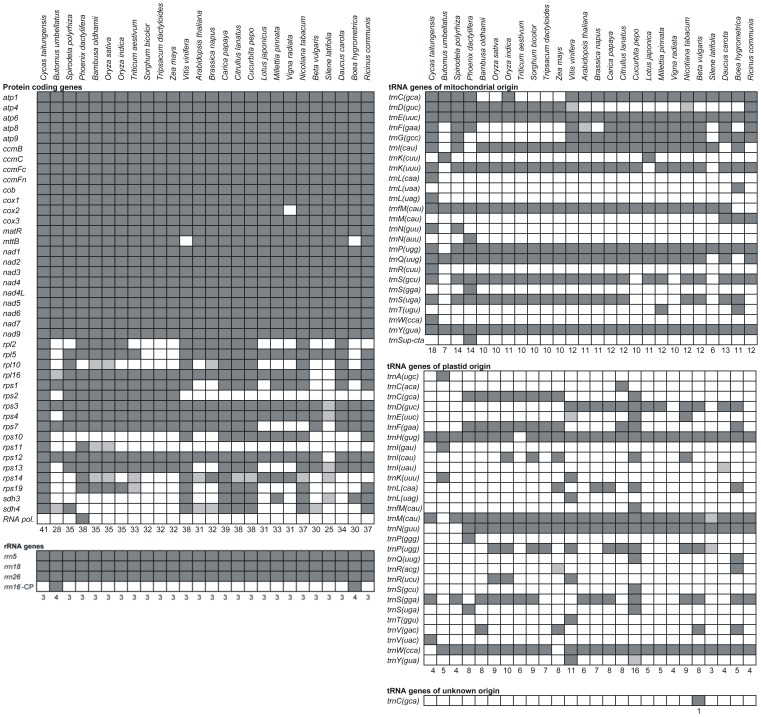
Gene content in selected seed plant mitochondrial genomes. Dark squares indicate presence of assumed intact, functional genes, lighter gray squares indicate putative pseudogenes, and white squares indicate absence of genes. Numbers below columns are the total numbers of assumed intact, functional genes. The table is modified and expanded from Sloan et al. [2].

The mitochondrial genome of *Butomus* contains 28 protein coding genes (Table 2, Fig. 2). Eighteen genes encode proteins of the respiratory chain (nine NADH dehydrogenase genes, five ATP synthase genes, three cytochrome C reductases, one cytochrome C reductase) and four are involved in cytochrome C biogenesis. Together with *matR* and *mttB* these 24 genes are almost ubiquitously found in all complete seed plant mitochondria sequenced so far. Exceptions are the loss of *cox2* in members of Fabaceae [36], of *mttB* in *Vitis* [37] and *Boea* [15], of a functional copy of *ccmFc* in *Silene conica* [2], and the potential loss of *atp8* in *Allium* [38].

Two succinate dehydrogenase genes, *sdh3* and *sdh4*, are present in the mitochondrial genome of *Cycas*, but based on Southern hybridization Adams et al. [39] suggested that these genes were lost repeatedly among the angiosperms, and in some cases transferred to the nucleus [38]. Among the Alismatales, data from Adams et al. [39] indicated presence of both genes in three genera of Araceae, and absence in two genera of Hydrocharitaceae and one genus of Alismataceae. Thus, the absence of both genes in *Butomus* is not surprising, though we have identified a partial, 157 bp long, pseudogene-like sequence of *sdh*4. In the completely sequenced mitochondrial genome of *Spirodela* (Araceae) *sdh4* is present as suggested by Adams et al. [39], but *sdh3* is not.

Only four ribosomal protein genes, *rps1*, *rps3*, *rps7*, and *rps12*, are found in the mitochondrial genome of *Butomus*. These genes are only rarely lost among angiosperms (Fig. 2) though Adams et al. [38] did report loss of *rps7* in the Hydrocharitaceae and Alismataceae. A lower number of ribosomal genes have so far only been recorded for species of *Silene* [2]. In *Spirodela*, no less than 10 functional ribosomal genes were detected. In addition to the four complete ribosomal protein genes, the *Butomus* mitochondrial genome includes a 398 bp long, pseudogene-like sequence of *rpl16*. Adams et al. [38] did not detect *rpl16* in the families Hydrocharitaceae and Alismataceae, both close relatives of *Butomus*.

The exact boundaries of less conserved protein coding genes may not be precisely determined neither in the present nor in previous investigations, where transcriptomes have not been studied. In addition to sequence variation, RNA editing which may affect all codons including start and stop codons potentially complicates precise assignments further. Accordingly, identification of the stop codon of *rps1* in the *Butomus* genome may be considered dubious. The 3′-end of the gene has little similarity to other *rps1* sequences and the designated stop codon is 19 bp inside a region supposed to be of plastid origin. Provided that the assignment of the gene and its stop codon, as well as the borders of the plastid insert region, are correct, it is possible that the plastid region has been inserted in the 3′-end of the gene thereby adding new amino acids and a new stop codon to the original *rps1* gene. However, it may be more probable that the true stop codon is located upstream of the currently recognized stop codon, but is not being recognized as such due to RNA editing.

### rRNA Genes

The mitochondrial genome of *Butomus* includes three rRNA genes (5S, 18S, and 26S rRNA genes) and as in all other mitochondrial genomes the 5S and 18S genes are located in very close proximity, whereas the larger 26S gene has a distant position (Fig. 1). In addition to the mitochondrial rRNA genes a copy of the plastid 16S rRNA gene is found as part of a 4897 bp fragment of plastid DNA (see above). A complete 16S rRNA gene has previously been observed in the mitochondrial genome of *Boea* [15] and a fragment was observed in *Cucumis* (GenBank acc. no. JF412792). In *Silene latifolia* and *S. vulgaris* Sloan et al. [9] described a possible gene conversion event between a mitochondrial 18S rRNA gene and a plastid 16S rRNA gene. The evidence for conversion was a ca. 50 bp segment of the mitochondrial 18S rRNA gene showing substantially more similarity to plastid 16S rRNA genes than to other mitochondrial 18S rRNA gene. As the mitochondrial genome of *Silene* does not include a plastid 16S rRNA gene or any substantial fragments of it, Sloan et al. [9] hypothesized that the originally transferred plastid gene has been lost from the mitochondrial genome. The plastid 16S rRNA gene found in *Butomus*, is very likely a result of a recent transfer event (see above). Repeated transfer and incorporation into the mitochondrial genome of 16S rRNA is supported by the two occurrences of partial or complete 16S rRNA sequences in *Cucumis* and *Boea*, Thus lending indirect support to the hypothesis of gene conversion in *Silene*.

### tRNA Genes

None of the angiosperm mitochondrial genomes sequenced to date has included a full set of tRNA genes, and genome of *Butomus* is no exception. Only 12 different tRNA genes could be found (Table 2, Fig. 2) and five of them (*trnA(ugc)*, *trnH(gug)*, *trnI(gau)*, *trnK(ugg)*, *trnW(cca)*) may be of plastid origin. The *trnH(gug)* gene has been indicated to be of plastid origin in all previously sequenced angiosperm mitochondrial genomes (except *Triticum*) as well as in *Cycas* [40], thus the inferred transfer event appear ancient. In contrast, both *trnA(ugc)* and *trnI(gau)* are located in a fragment of plastid DNA which also includes 16S rRNA and apparently represents a very recent transfer (see above). This is consistent with the two tRNA genes being absent from all other mitochondrial genomes sequenced so far. As the only tRNA genes found in *Butomus*, the latter two contain introns of 684 bp (*trnA(ugc)*) and 937 bp (*trnI(gau)*), respectively.

With the exception of *Zea* all complete mitochondrial genomes of angiosperms seem to include a copy of *trnW(cca)* similar to the corresponding plastid gene, whereas *Cycas* has a copy of *trnW(cca)* more similar to the mitochondrial versions of the gene found in e.g., mosses, liverworts, algae, etc. Consistent with a plastid origin, we find the *trnW(cca)* gene in a larger region apparently of plastid origin and shared by many angiosperms (see above). Thus, data suggests that the transfer took place either in the early evolution of the angiosperms or even prior to that. The *trnK(uuu)* gene of *Butomus* is included in the same fragment, potentially of plastid origin, as *trnW(cca)* (see above), but in plastid genomes the >90% similar sequence encodes *trnP(ugg).* This clearly illustrates the ambiguity in trying to determine the homology of individual tRNA genes. Due to high sequence similarity between many tRNA genes and the automated naming of the gene, which may change due to a single base change in the anticodon region, similar named genes may not be homologous whereas differently named genes, such as here *trnK(uuu)* and *trnP(ugg)*, may be truly homologous. Here we list *trnK(uuu)* as of plastid origin (Table 2, Fig. 2), but the sequence may be homologous to sequences either listed as mitochondrial *trnP’*s, *trnK’*s, or even other genes in other studied mitochondrial genomes. The wobble pairing mechanism, which further introduces ambiguity in codon-anticodon recognition [41], makes naming and prediction of tRNAs even more complicated when only raw sequence information is available. Thus, the tRNA gene content listed in figure 2 and similar tables in other paper e.g., [9], [42], need not reflect functionality or homology precisely, and the distinction between tRNA genes of plastid and mitochondrial origin is fuzzy and should be considered with great caution.

### Introns

In *Butomus* we found a total of 21 group II introns distributed among seven protein coding genes (Table S1). Most of the introns are *cis*-spliced, but five are *trans*-spliced (two in *nad1*, one in *nad2*, and two in *nad5*). The *cis*-spliced introns include a total of 23,946 bp corresponding to ca. 5% of the mitochondrial genome of *Butomus*. Compared to the overview of intron content of selected angiosperms provided by Mower et al. [30], *Butomus* includes the same 12 *cis*-spliced and five *trans*-spliced introns indicated as universally present among angiosperms. The second intron of *nad4* and the third intron of *nad7*, which are not universally present, are both found in the *Butomus* genome and so far they appear to be present in all monocotyledons. The fourth intron of *nad1* which varies between being *cis*- and *trans*-spliced, is *cis*-spliced in *Butomus*, whereas it is *trans*-spliced in grasses, *Phoenix*, and some eudicotyledons [30], [42]. We interpret the intron to be *trans*-spliced in *Spirodela*, even though there are discrepancies between the figure of the mitochondrial genome (Figure 3 in [14]), a table of genes (Table S3 in [14]), and the GenBank record (NC_017840). Compared to the *Butomus* mitochondrial genome and the genomes reviewed by Mower et al. [30] *Spirodela* appears to lack the third of the five exons of *nad5* [14]. However, this exon is very short (usually just 21–22 bp) and easily overlooked, but a BLAST search reveals its presence in *Spirodela* with the flanking introns both being trans-spliced. With these corrections regarding *nad1* and *nad5*, the total number of introns (21) and the distribution of *cis*-spliced (15) and *trans*-spliced (6) introns given by Wang et al. [14] becomes correct, though tables, figures, and the GenBank file all seem to contain errors.

**Figure 3:**
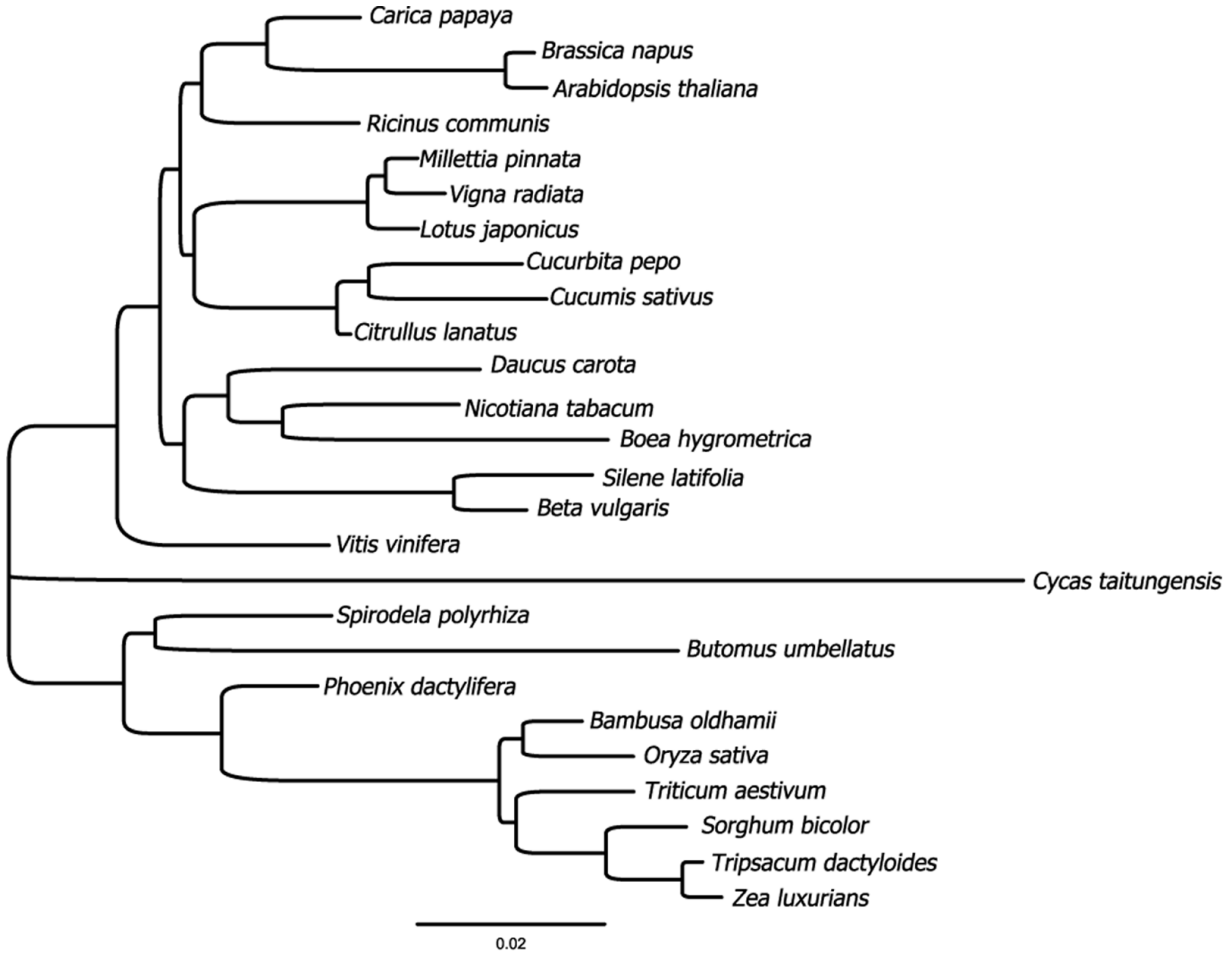
Phylogeny of 26 seed plants inferred from 24 protein coding mitochondrial genes. Phylogenetic tree based on Maximum Likelihood with branch lengths proportional to substitution per site.

In *cox2*, most angiosperms have either one or more rarely two introns (see [30]), but *Butomus* lacks both these introns. As in many other plant mitochondria, *matR* is located within intron 4 of *nad1*. The group I intron found in *cox1* of many angiosperms is not present in *Butomus* consistent with its reported lack in three species of core alismatids, including two species of Hydrocharitaceae, the sister group to *Butomus* [43].

In addition to the introns located in protein coding genes, we found one intron in each of two tRNA genes, *trnA(ugc)* and *trnI(gau)*, both presumably transferred from the plastid genome (see above).

### Repetitive Sequences

Repetitive sequences, often of considerable length, are common in plant mitochondria; the longest being a perfect 87 kb repeat found in *Beta* [44]. Differences in overall mitochondrial genome size can to some extent be explained by presence of such repeats [7].

In *Butomus* we found one long, direct repeat of ca. 9 kb (RR1; Table 4, Fig. 1), and two shorter, inverted repeats of 6.3 kb and 2.8 kb, respectively (RR2 and RR3; Table 4, Fig. 1). Additionally, 40 short repeats (from 50 bp to 1 kb) were identified ranging in copy numbers from two to five (Table 4 [L>200], Table S2 [50<L<200]). Repeats shorter than 50 bp are not listed. The repeated sequences cover approximately 37.8 kb (8.3%) of the mitochondrial genome of *Butomus*. However, some of the repeats are overlapping and the entire fraction of the mitochondrial genome covered by repeated sequences is less than the sum of lengths of all repeat fragments. The amount of repeats and their contribution to genome size falls within the range found in other angiosperm genomes, where the lowest content is 2.3% found in *Phoenix* and the highest 36.4% found in *Tripsacum* [42].

**Table 4:** Long repeats in the mitochondrial genome *of Butomus umbellatum.*

Repeat	Size (kb)	% Identity	E value	Directionality
RR1	9.0	100	0	+/+
RR2	6.3	100	0	+/−
RR3	2.8	100	0	+/−
RR4	0.75	97	0	+/+/+/−
RR5	0.4	100	0	+/−
RR6	0.3	88	1.00×10^−92^	+/−
RR7	0.25	96	1.00×10^−113^	+/+
RR8	0.25	100	4.00×10^−128^	+/+
RR9	0.25	100	6.00×10^−126^	+/+
RR10	0.2	88	1.00×10^−57^	+/−

Only repeats >0.2 Kb and identity score >80% are shown. RR1 to RR10 refer to the nomenclature used in Fig. 1. RR4 has four copies, the remaining has only two. Direct and inverted repeats are indicates as+and –.

Some of the repeated sequences in *Butomus* include entire genes, which thus occur in duplicate or triplicate. The protein coding *ccmC* gene occurs in three identical copies, and the tRNAs *trnE(uuc)* and *trnH(gug)*, occur in three and two identical copies, respectively.

In particular longer repeats, i.e. >1 kb, are thought to mediate homologous recombination [6], but to what extent repeats found in *Butomus* are involved in recombination is unknown.

### RNA Editing

In all land plants (Embryophytes), except marchantiid liverworts, transcripts of the mitochondrial genome are known to experience extensive RNA editing, primarily as C-to-U changes [45], [46]. Editing mostly affects protein coding gene, breaking the normal 1∶1 correspondence between DNA sequence and amino acid sequence of the protein, but non-protein coding sequences, e.g., introns and tRNAs may also be edited (e.g., [47] and references therein).

The exact number of edited positions in a complete genome is rarely known. This is primarily due to lack of transcriptome data, but also due to the only partial or tissue specific nature of editing [48]. In the best studied angiosperm genomes the total number of edited sites (in the protein coding genes) range from ca. 200–500 positions [7], [8], [33], [48]–[51]. In *Phoenix* and *Spirodela* approximately 600 sites are estimated to be edited [14], [42], in *Amborella* an unpublished survey revealed 835 edited sites [4], and in the only non-angiosperm seed plant investigated to date, *Cycas*, more than 1000 sites were estimated to be edited [40]. Using PREP-Mt [52], [53] we estimate that in *Butomus* 28 protein coding genes include 557 edited sites.

In an earlier study of selected mitochondrial genes in alismatids we experimentally verified RNA editing in partial *atp1* and *ccmB* sequences of *Butomus* [18]. Compared to the predictions made here, we found two additional edited sites in *atp1*. In *ccmB* we found six extra edited sites, but PREP-Mt predicted a further six sites to be edited, which were actually not edited. Thus, the PREP-Mt predictions occasionally slightly underestimate the actual number of edited sites. Despite using a higher cut-off values than us (0.6 vs. 0.2), thereby lowering the number of predicted edited sites, Wang et al. [14] report that PREP-Mt may potentially produce almost 10% erroneous predictions. However, they do not report the number of edited sites found only by comparisons to RT-PCR sequences of the four investigated genes, i.e. they report only false positives, not false negatives. Regardless, the 557 sites predicted to be edited in *Butomus*, should not be considered exact, but the figure is in line with the range found in other genomes.

All protein coding genes in *Butomus* are estimated to have edited sites. In some genera of the Alismataceae and the Hydrocharitaceae previous studies have shown that some mitochondrial genes lack editing completely [17], [18]. It was suggested that those genes originated through retrotranscription (“processed paralogs”) and in a number of core alismatids (but not in *Butomus*) two copies of *nad1*, one including and one excluding edited sites, respectively, were found [19]. Even though these earlier studies have suggested that the patterns of evolution of both the genes and RNA editing in the alismatids are in need of further exploration, present data from *Butomus* confirm absence of aberrant traits in this taxon.

### Phylogeny and Substitution Rates

As expected, a phylogenetic analysis of 24 protein coding genes from 25 angiosperms and *Cycas* places *Butomus* as sister to *Spirodela* (Fig. 3) in accordance with previous phylogenetic studies [54]. Despite the poor taxon sampling the tree is generally in good agreement with the current phylogenies of the angiosperms (see http://www.flmnh.ufl.edu/angiospermATOL/index.html). At family level only the positions of *Vitis* and *Ricinus* differ from current views, but within the three families represented by more than two genera only the relationships within Fabaceae is resolved as expected [55], whereas the relationships within Cucurbitaceae is not [56], and the resolution within Poaceae is only partially so [57].

Compared to *Spirodela* the overall substitution rate of the protein coding genes of *Butomus* is significantly increased and a similar difference in rates can be seen between *Phoenix* and the grass clade (Fig. 3). Based on data from individual mitochondrial genes Petersen et al. [17] and Cuenca et al. [18] have previously found a highly elevated substitution rate in the core alismatids in comparison not only to members of the Araceae, but to most monocotyledons, and palms were found to have a very low substitution rate. The substitution rate difference between *Butomus* and *Spirodela* differs for individual protein coding genes, but we consistently find a higher rate in *Butomus* (data not shown).

**Figure 4:**
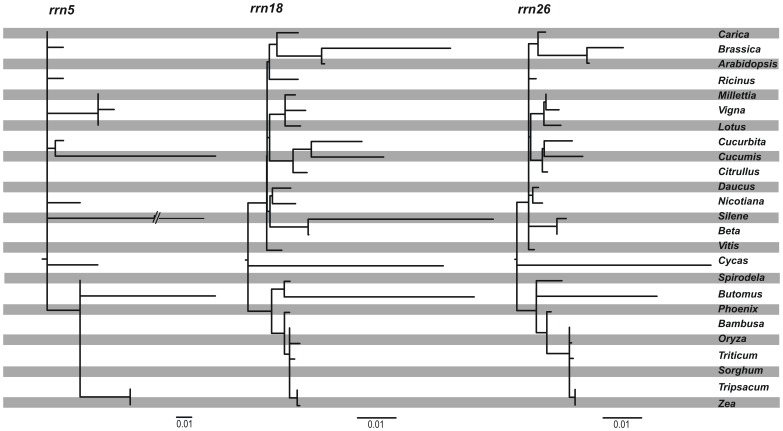
Phylogenetic analysis of substitution rates in three mitochondrial rRNA genes. Analyses are based on a constraint tree using the topology from analysis of 24 protein coding genes (Fig. 3). Branch lengths are proportional to substitutions per site except the *Silene* branch in the *rrn5* tree having been reduced to ca. 1/3 of its actual length.

Sloan et al. [9] investigated substitution rates of mitochondrial ribosomal genes and discovered an extremely elevated substitution rate of the 5S rRNA gene and a moderately elevated substitution rate of the 18S rRNA gene in *Silene latifolia*. In *Butomus* we observe an elevated but not significantly different substitution rate of all three rRNA genes compared to the genes of all other monocotyledons, but not to all core eudicotyledons (Fig. 4).

### Perspectives

The number of complete plant mitochondrial genomes is growing rather slowly, primarily due to their complex and labile structure, large amounts of repeats, and their alleged ability to accept alien DNA sequences both through intracellular (viz. from the nucleus and plastids) and horizontal gen transfer (viz. transgressing species boundaries). A potential further complication is RNA editing and the occurrence of processed paralogs.

The limited number of available mitochondrial plant genomes in GenBank is in stark contrast to the number of complete plastid genomes and animal mitochondrial genomes present. However, the majority of animal mitochondrial genomes and plastid genomes are characterized by sharing a rather monotone size and structure, similarities clearly reflected in the number of organelle genomes in GenBank, thus as of December 3^rd^ 2012 there are 72 complete plant (only 41 from Spermatophyta) and 2831 complete metazoan mitochondrial genomes and 223 complete plastid genomes (120 from Spermatophyta).

The vast majority of the sequenced plant mitochondrial genomes are from commercially important crop species and few are selected for their phylogenetic importance. This bias (e.g. of the 40 sequenced genomes from angiosperms, 10 genomes are from grasses) evidently impair our ability to acquire an in-depth understanding of key evolutionary issues such as; how much gene loss is tolerated in the mitochondria of photosynthetic plants or in plants showing different levels of parasitism (e.g. from facultative parasitism to holo-parasitism)? It also weakens our ability to obtain solid evidence for HGT, which is primarily based on conflicts in phylogenetic evidence [58].

The relatively simple nature of animal mitochondria and plant plastids made them ideally suited for extracting phylogenetic information, and sequences from these – usually maternally inherited – organelles have dominated animal and plant phylogeny for two decades. This is most likely not going to change over-night, but these phylogenies will increasingly be challenged by genomic data, from the nucleus, and in plants also from the mitochondrion.

## Supporting Information

Table S1 **Introns in mitochondrial genes of** ***Butomus umbellatus.***
(DOCX)

Table S2 **Repeated sequences >50 bp in the mitochondrial genome of** ***Butomus umbellatus.***
(DOCX)

Table S3 **Predicted edited sites in protein coding genes in the mitochondrial genome of** ***Butomus umbellatus*** **.**
(DOCX)
