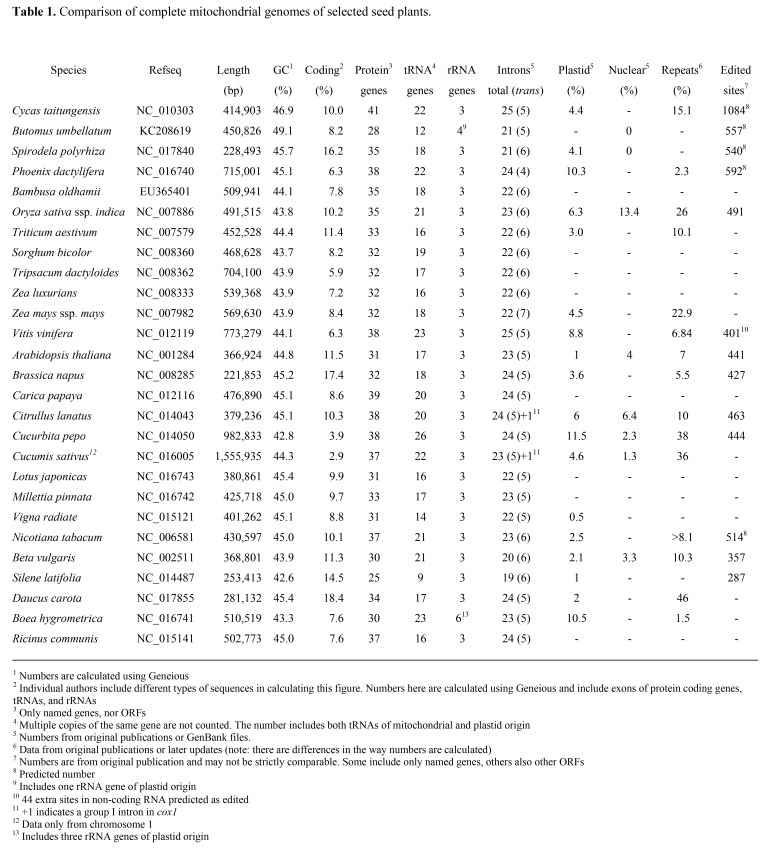# Correction: The Complete Sequence of the Mitochondrial Genome of *Butomus umbellatus* – A Member of an Early Branching Lineage of Monocotyledons

**DOI:** 10.1371/annotation/024aa8f2-a9cb-46a7-93ce-5a193189dea9

**Published:** 2013-10-16

**Authors:** Argelia Cuenca, Gitte Petersen, Ole Seberg

Due to issues with the typesetting process, there were errors in Table 1. The correct version of the table is available here: 

**Figure:**