# Supplementary material for: The Complete Sequence of the Mitochondrial Genome of Butomus umbellatus – A Member of an Early Branching Lineage of Monocotyledons
Source: PLoS One. 2013 Apr 24;8(4):e61552. doi: 10.1371/journal.pone.0061552 (PMC3634813; doi:10.1371/journal.pone.0061552)
Supplement: Table S1 — Introns in mitochondrial genes of Butomus umbellatus. (DOCX) [file pone.0061552.s001.docx]

**Table S1.** Introns in mitochondrial genes of *Butomus umbellatus.*

| **Intron** | **start** | **end** | **length (if *cis*-spliced)** |
| --- | --- | --- | --- |
| *nad1i1* | 250601F | 261782R | *trans*-spliced |
| *nad1i2* | 260812 | 261782 | 971 |
| *nad1i3* | 260812R | 27779R | *trans*-spliced |
| *nad1i4* | 23616 | 27839 | 4224 (including *matR*) |
| *nad2i1* | 84763 | 85580 | 818 |
| *nad2i2* | 85973F | 50877F | *trans*-spliced |
| *nad2i3* | 50879 | 52530 | 1652 |
| *nad2i4* | 53104 | 54451 | 1348 |
| *nad4i1* | 355669 | 356613 | 945 |
| *nad4i2* | 357129 | 360532 | 3404 |
| *nad4i3* | 360956 | 362561 | 1606 |
| *nad5i1* | 267203 | 268042 | 840 |
| *nad5i2* | 265991R | 11156 | *trans*-spliced |
| *nad5i^1^* | 11178 | 430531 | *trans*-spliced |
| *nad5i4* | 429221 | 430138 | 918 |
| *nad7i1* | 449512 | 450389 | 878 |
| *nad7i2* | 448114 | 449442 | 1329 |
| *nad7i3* | 446684 | 447646 | 963 |
| *nad7i4* | 444770 | 446439 | 1670 |
| *ccmFci1* | 233270 | 234791 | 1522 |
| *rps3i1* | 6921 | 8116 | 1196 |
| *trnI(gau)i1* | 370033 | 370952 | 920 |
| *trnA(ugc)i1* | 371107 | 371790 | 684 |

The position and length of introns in eight protein coding and two tRNA genes in the mitochondrial genome of *Butomus umbellatus*. Length is only given for *cis*-spliced introns.
