# Supplementary material for: The Complete Sequence of the Mitochondrial Genome of Butomus umbellatus – A Member of an Early Branching Lineage of Monocotyledons
Source: PLoS One. 2013 Apr 24;8(4):e61552. doi: 10.1371/journal.pone.0061552 (PMC3634813; doi:10.1371/journal.pone.0061552)
Supplement: Table S2 — Repeated sequences >50 bp in the mitochondrial genome of Butomus umbellatus. (DOCX) [file pone.0061552.s002.docx]

**Table S2**. Repeated sequences >50bp in the mitochondrial genome of *Butomus umbellatus.*

| **Repeat** | **% identity** | **Length** | **Copy 1** | **Copy 2** | **Copy 3** | **Copy 4** | **Copy 5** | **Expected value** | **Bit score** | **Directionality** |
| --- | --- | --- | --- | --- | --- | --- | --- | --- | --- | --- |
| RR1 | 99,98 | 9024, 9025 | 56657 | 294098 |  |  |  | 0 | 1,67×10^4^ | +/+ |
| RR2 | 99,98 | 6329 | 62908 | 360879 |  |  |  | 0 | 1,17×10^4^ | +/- |
| RR3 | 99,96 | 2773 | 300350 | 364435 |  |  |  | 0 | 5118 | +/- |
| RR4 | 97.00-97.13 | 749, 766 | 64568 | 302010 | 333405 | 364782 |  | 0 | 1271-1277 | +/+/+/- |
| RR5 | 100 | 376 | 414827 | 444336 |  |  |  | 0 | 695 | +/- |
| RR6 | 88,16 | 292, 295 | 381766 | 433950 |  |  |  | 1,00×10^-92^ | 342 | +/- |
| RR7 | 95,77 | 252, 260 | 1002 | 268240 |  |  |  | 1,00×10^-113^ | 412 | +/+ |
| RR8 | 100 | 249 | 134430 | 355103 |  |  |  | 4,00×10^-128^ | 460 | +/+ |
| RR9 | 100 | 245 | 92669 | 262862 |  |  |  | 6,00×10^-126^ | 453 | +/+ |
| RR10 | 87,5 | 190, 216 | 243259 | 407069 |  |  |  | 1,00×10^-57^ | 226 | +/- |
| RR11 | 98,91 | 183 | 378503 | 418987 |  |  |  | 4,00×10^-88^ | 327 | +/- |
| RR12 | 100 | 173 | 243446 | 393912 |  |  |  | 6,00×10^-86^ | 320 | +/+ |
| RR13 | 98,25 | 171 | 336642 | 414284 |  |  |  | 8,00×10^-80^ | 300 | +/- |
| RR14 | 85,54 | 158, 160 | 2878 | 29674 |  |  |  | 4,00×10^-38^ | 161 | +/- |
| RR15 | 97,97 | 147, 148 | 83669 | 243128 |  |  |  | 2,00×10^-66^ | 255 | +/+ |
| RR16 | 85,71 | 143, 151 | 382160 | 433797 |  |  |  | 9,00×10^-35^ | 150 | +/- |
| RR17 | 86,13 | 129, 135 | 11987 | 247932 |  |  |  | 2,00×10^-31^ | 139 | +/+ |
| RR18 | 98,37 | 122 | 6786 | 329120 |  |  |  | 3,00×10^-54^ | 215 | +/- |
| RR19 | 99,15 | 118 | 262162 | 367813 |  |  |  | 1,00×10^-53^ | 213 | +/- |
| RR20 | 82,5 | 111, 117 | 280125 | 402273 |  |  |  | 4,00×10^-18^ | 95,3 | +/+ |
| RR21 | 99,04 | 103, 104 | 38935 | 66199 | 363814 |  |  | 2,00×10^-45^ | 185 | +/-/+ |
| RR22 | 85,58 | 98, 102 | 83947 | 92918 |  |  |  | 2,00×10^-20^ | 102 | +/- |
| RR23 | 92,78 | 96,97 | 246638 | 288238 |  |  |  | 2,00×10^-31^ | 139 | +/- |
| RR24 | 97,87 | 92, 94 | 61856 | 160087 | 299297 |  |  | 4,00×10^-38^ | 161 | +/-/+ |
| RR25 | 98,89 | 90 | 34750 | 373585 |  |  |  | 4,00×10^-38^ | 161 | +/- |
| RR26 | 95,56 | 89 | 23318 | 328218 |  |  |  | 1,00×10^-32^ | 143 | +/+ |
| RR27 | 100 | 83 | 39202 | 250147 |  |  |  | 5,00×10^-37^ | 158 | +/+ |
| RR28 | 100 | 81 | 238268 | 424610 |  |  |  | 9,00×10^-35^ | 150 | +/+ |
| RR29 | 90,67 | 71,75 | 81919 | 258231 |  |  |  | 1,00×10^-28^ | 97,1 | +/- |
| RR30 | 98,63 | 73 | 84013 | 243609 |  |  |  | 1,00×10^-28^ | 130 | +/+ |
| RR31 | 97,1 | 69 | 12195 | 413355 |  |  |  | 9,00×10^-25^ | 117 | +/+ |
| RR32 | 86,36 | 62, 66 | 206808 | 372470 |  |  |  | 3,00×10^-10^ | 69,4 | +/+ |
| RR33 | 93,75 | 62, 64 | 13001 | 413706 |  |  |  | 4,00×10^-18^ | 95,3 | +/+ |
| RR34 | 96,83 | 63 | 7529 | 267615 |  |  |  | 2,00×10^-21^ | 106 | +/- |
| RR35 | 93,65 | 63 | 28513 | 65112 | 302554 | 333932 | 364941 | 4,00×10^-18^ | 95,3 | +/+/+/+/- |
| RR36 | 95,08 | 60, 61 | 136127 | 170381 |  |  |  | 4,00×10^-18^ | 95,3 | +/+ |
| RR37 | 96,67 | 60 | 238348 | 424755 |  |  |  | 9,00×10^-20^ | 100 | +/+ |
| RR38 | 98,28 | 57, 58 | 85965 | 243683 |  |  |  | 9,00×10^-20^ | 100 | +/+ |
| RR39 | 91,53 | 55, 59 | 63954 | 301396 | 366103 | 413006 |  | 4,00×10^-13^ | 78,7 | +/+/-/- |
| RR40 | 100 | 53 | 274565 | 388483 |  |  |  | 3,00×10^-19^ | 99 | +/+ |
| RR41 | 100 | 52 | 24095 | 47495 |  |  |  | 1,00×10^-18^ | 97,1 | +/- |
| RR42 | 92,16 | 51 | 117000 | 448712 |  |  |  | 2,00×10^-11^ | 73,1 | +/- |
| RR43 | 90,2 | 50, 51 | 372080 | 418422 |  |  |  | 3,00×10^-9^ | 65,8 | +/- |

List of all repeated sequences >50 bp and with a similarity >80%. For each repeat copy the first position is listed. Directionality of repeats is indicated as + for direct repeats and – for inverted repeats. The repeats are sorted according to average length.
