# Supplementary material for: The Complete Sequence of the Mitochondrial Genome of Butomus umbellatus – A Member of an Early Branching Lineage of Monocotyledons
Source: PLoS One. 2013 Apr 24;8(4):e61552. doi: 10.1371/journal.pone.0061552 (PMC3634813; doi:10.1371/journal.pone.0061552)
Supplement: Table S3 — Predicted edited sites in protein coding genes in the mitochondrial genome of Butomus umbellatus . (DOCX) [file pone.0061552.s003.docx]

Table S3. Predicted edited sites in protein coding genes in the mitochondrial genome of *Butomus umbellatus*.

| **Gene** | **Gene length** | **No. edited sites^1)^** | **% edited sites** |
| --- | --- | --- | --- |
| *nad1* | 978 | 27 | 2.8 |
| *nad2* | 1467 | 31 | 2.1 |
| *nad3* | 357 | 18 | 5.0 |
| *nad4* | 1488 | 52 | 3.5 |
| *nad4L* | 303 | 14 | 4.6 |
| *nad5* | 1992 | 33 | 1.7 |
| *nad6* | 690 | 16 | 2.3 |
| *nad7* | 1182 | 34 | 2.9 |
| *nad9* | 618 | 12 | 1.9 |
| *cob* | 1179 | 16 | 1.4 |
| *cox1* | 1584 | 25 | 1.6 |
| *cox2* | 669 | 15 | 2.2 |
| *cox3* | 798 | 15 | 1.9 |
| *atp1* | 1470 | 8^2)^ | 0.5 |
| *atp4* | 606 | 9 | 1.5 |
| *atp6* | 915 | 22 | 2.4 |
| *atp8* | 480 | 5 | 1.0 |
| *atp9* | 225 | 2 | 0.9 |
| *ccmB* | 621 | 39 | 6.2 |
| *ccmC* | 780 | 31 | 4.0 |
| *ccmFc* | 1326 | 17 | 1.3 |
| *ccmFn* | 831 | 37 | 4.5 |
| *matR* | 2027 | 18 | 0.9 |
| *mttB* | 843 | 32^3)^ | 3.8 |
| *rps1* | 558 | 4 | 0.7 |
| *rps3* | 1590 | 16 | 1.0 |
| *rps7* | 432 | 3 | 0.7 |
| *rps12* | 378 | 6 | 1.6 |
| **Total** | **26387** | **557** | **2.1** |

^1)^ Number of edited sites estimated using PREP-Mt with a cutoff value C >0.2

^2)^ In a mRNA sequence of 906 bp we found two extra edited sites [18]

^3)^ In a mRNA sequence six extra edited sites are found, but six sites predicted as edited by PREP-Mt are not edited [18]
